# Paternal Dietary Methionine Supplementation Improves Carcass Traits and Meat Quality of Chicken Progeny

**DOI:** 10.3390/ani11020325

**Published:** 2021-01-28

**Authors:** Mohamed Shafey Elsharkawy, Ying Chen, Ranran Liu, Xiaodong Tan, Wei Li, Ibrahim El-Wardany, Dongqin Zhao, Maiqing Zheng, Jie Wen, Guiping Zhao

**Affiliations:** 1 Institute of Animal Sciences, Chinese Academy of Agricultural Sciences, Beijing 100193, China; shafey.nrc@hotmail.com (M.S.E.); chenying1482@163.com (Y.C.); liuranran112@126.com (R.L.); tanxiaodong08@163.com (X.T.); liweirfi@126.com (W.L.); shlo_57127737@163.com (D.Z.); zhengmaiqing@caas.cn (M.Z.); wenjie@caas.cn (J.W.); 2 State Key Laboratory of Animal Nutrition, Beijing 100193, China; 3 Animal Production Department, National Research Centre, Dokki, 12622 Cairo, Egypt; 4 Poultry Production Department, Faculty of Agriculture, Ain Shams University, 11241 Cairo, Egypt; ielwardany2010@yahoo.com

**Keywords:** methionine, carcass traits, paternal effects, progeny, meat quality, gene expression

## Abstract

**Simple Summary:**

High meat quality is one of the demands made by consumers. Therefore, studies have concluded that meat quality can be improved through some feed additives to broiler diets. Other studies showed that broilers meat quality improved by adding some nutrients to the maternal diet, so this study was conducted to evaluate the paternal effects of dietary 0.1% methionine on meat quality and carcass traits of offspring. Our results suggested that paternal dietary supplementation with 0.1% methionine enhances offspring carcass eviscerated yield, thigh muscles yield and reduces abdominal fat, also meat shear force, drip loss, pH-value, and color. The expression of genes associated with meat quality support these results.

**Abstract:**

The effects that maternal dietary methionine have on progeny have been reported on broilers. However, the paternal effects are not known, so the current study was conducted to explore the influences of paternal dietary methionine (Met) have on progeny carcass traits, meat quality, and related gene expressions. A total of 192 hens and 24 roosters from Ross parent stock at 36 weeks of age were selected. From week 37 to 46, the roosters were allocated to two groups with three replicates of 4 cocks each, (control, 0.28% Met), and methionine group (MET group, 0.28% Met + 0.1% coated Met). The results revealed that, although the heavier live body weight in progeny at day 49 of control group compared to MET group (*p* < 0.05), the relative eviscerated yield and relative thigh muscle yield were higher in MET group (*p* < 0.05); but the relative abdominal fat was lower (*p* < 0.05). In thigh and breast muscles, a positive response of pH_24 h_ value, shear force (g) and drip loss (%) were observed in MET group (*p* < 0.05). The lightness (L) and redness (a) were increased (*p* < 0.05) in breast muscles of MET group, while only the redness (a*_24 h_) and yellowness (b*_24 h_) were increased (*p* < 0.05) in thigh muscles of MET group. The gender has a significant (*p* < 0.05) effect on carcass traits and muscle redness (a*), where these traits improved in males, and no interaction between treatments and gender were observed for these results. The expression levels of *PRKAG2* and *PRDX4* supported the changes in muscle pH, with these up-regulated in thigh and breast muscles of MET group, the *PPP1R3A* gene supported the changes in pH value being down-regulated (*p* < 0.01) in these same muscles. The *BCO1* gene expression was consistent with the changes in meat color and was up-regulated (*p* < 0.01) in thigh muscles of MET group, consistent with the changes in b* color values. Finally, it was concluded that the supplementation of 0.1% Met to rooster diets could improve carcass characteristics and meat quality of progeny.

## 1. Introduction

Continuous genetic selection, has resulted in fast growing broilers that reach market weight by six weeks of age with high meat yield [1]. The selection has generated adverse effects on the meat quality, by increasing fiber diameters and the ratio of glycolytic fibers [2]. The quality of meat is an intricate feature affected by environmental, genetic and nutritional variables [3], so several studies have evaluated the influence of chickens nutrient supplementation on meat quality [4,5,6].

For corn-based broiler diets, methionine (Met) is considered the first limiting specific amino acid for synthesis of protein and the source of S-adenosylmethionine, the donor of methyl group for DNA methylation [7,8,9]. A methyl group provided by S-adenosylmethionine is required for the biosynthesis of epinephrine, choline, carnitine, and creatine, which are essential for muscle development [10,11]. Methionine enhances growth performance and feed efficiency of broilers [11,12]. Previous studies proved that many feed additives, including vitamins [13], minerals [14], or the presence of toxins [15] in laying hen diets have a trans-generational effect on progeny performance. There are reports identifying the influence of maternal nutrition of laying hens on meat quality, growth and disease resistance of progeny [14,16,17]. Recently, some reports studied the effect of maternal dietary Met on the chicken progeny [16], ducks [17], and Japanese quail [18].

There are studies indicating that the dietary Met improves productive performance [6] and enhance carcass traits in chicken [19,20,21]. Meat quality of broilers, such as pH and meat color were improved by dietary Met [22]. Coated Met has been used widely in ruminant and aquatic nutrition because being protected it has a higher absorption rate [23,24]. The maternal effects of dietary Met in poultry diets on performance, quality of meat and carcass characteristics of progeny has been reported and it was shown that 0.1% coated Met was an adequate inclusion rate [16].

Roosters have an essential role in the broiler breeding system, where they constitute 50% of the genetic make-up of progeny and the flock fertility [25]. Methionine is an essential nutrient that promotes rooster semen production and improves semen quality [26,27]. Still, there is a lack of knowledge about the transgenerational influence of nutrients to progeny through parents. Therefore, we assume that paternal dietary Met supplementation will have an influence on carcass traits and meat quality of the offspring. So, this study aimed to explore the transgenerational influences of paternal dietary Met on meat quality and carcass traits of progeny.

## 2. Materials and Methods

### 2.1. Animals and Experimental Diets

The use of animal in this research was approved by the Institute of Animal Sciences, Chinese Academy of Agricultural Sciences (IAS-CAAS, Beijing, China). A total Ross 192 hens and 24 roosters at 36 weeks of age obtained from Hebei Feilong Poultry Co., Ltd., (Xingtai, China). The roosters were randomly split into two groups (control group and methionine group) with three replicates of four roosters each. The control group was fed a basal diet with 0.28% endogenous Met, whereas the MET group were fed a diet with 0.38% Met, formulated as shown in Table 1 by adding 0.1% coated Met to the basal diet. The coated Met, containing 50% of an active substance was obtained from Hangzhou King Technology Feed Co., Ltd. (Hangzhou, China). The 192 hens also split into two groups with three replicates of 32 hens each; the two groups were fed a basal diet with 0.37% Met. All diets have been formulated according to (NRC, 1994) poultry nutrient requirements. Prenatal birds were raised on floor and, the feed and water were provided ad libitum for 10 weeks. The 32 hens and four roosters were housed in the same pen (Sexual ratio, 1:8) with separation of male and female diet, and the progeny were generated through random mating. After incubation and hatching, the progeny was raised in stair-step cages. The progeny from two groups with three replicates each fed the same basal diet formulated according to NRC (1994) to fulfill the nutrition requirement of Ross broilers. The feed and water were provided ad libitum for 49 days.

### 2.2. Samples Collection and Carcass Traits

At day 49, the progeny broilers (20 males and 23 females per replicate) were randomly selected, weighed, and slaughtered by cutting the jugular vein after fasted for 12 h. The eviscerated yield was measured as a live body weight percentage. The percentages of thigh and breast muscle, and abdominal fat weight were measured as an eviscerated carcass weight percentage. Samples of muscles (thigh and breast) were quickly snap-frozen in liquid nitrogen, then stored at −80 °C for subsequent analysis.

### 2.3. Meat Quality Traits

The breast and thigh muscles were stored at 4 °C after stripping. The meat color of thigh and breast muscles such as lightness (L), redness (a), and yellowness (b) (L*_45 min_, L*_24 h,_ a*_45 min_, a*_24 h_, b*_45 min_, b*_24 h_) were assessed at 45 min and 24 h after slaughter using a spectrophotometer (Konica Minolta CR 410) by the three-dimensional CIELAB color space system [28], which creates a color space based on mathematic calculations involving three independent variables: L*, a*, and b*, and permits quantitative color comparisons [29]. The pH-value of thigh and breast muscles were analyzed using a pH meter (HI8424, HANNA Instruments, Italy) with a thin penetrating tip inserted 0.5–1.0 cm below the surface of the muscle [30]. To analyze the drip loss, the muscles were trimmed, blotted to remove the surface water, and weighed. Samples were then placed in a plastic bag filled with air and fastened under the temperature of 4 °C, they were then weighed following a period of 24 h. Percentage of drip loss was calculated by 100 × (initial weight − final weight 24 h)/initial weight [31]. The shear force of samples was measured based on the mechanical force (g) required to shear the muscle fibers of a cooked meat sample [32] using Texture Analyzer (TA.XT Plus, Stable Micro System, Surrey, England). The samples of 2 cm × 1 cm × 1 cm were cooked in a pre-heated water bath set at 80 °C. The cooking lasted 10 min after a center temperature of the samples reached 75 ± 1 °C, then cooled to 4 °C. The equipment was calibrated at 5 kg for weight, 10 mm return distance for height and the blade speed was set at 10 mm/s.

### 2.4. RNA Isolation and Real-Time Quantitative qRT-PCR

All procedures of isolation, quantification, and reverse transcription of total RNA from muscles were conducted as reported earlier [33]. Briefly, the total RNA was extracted from frozen muscles using RNA Isolation Kit (TIANGEN, Beijing, China). After treatment with RNase-Free DNase I (New England Biolabs, Ipswich, MA, USA), total RNA was extracted with phenol-chloroform and precipitated with ethanol. The quality and concentration of the total RNA were determined with a NanoDrop 2000 spectrometer (Thermo Fisher Scientific, Waltham, MA, USA) and its integrity was further verified by agarose gel electrophoresis with RNA integrity number (RIN >  6). Total RNA (2.5 μg) from each sample was used to generate complementary DNA (cDNA) in a final volume of 20 μL according to the manufacturer’s instructions (Promega, Wisconsin, USA). The cDNA was then diluted 1:10 with nuclease-free water (Ambion, Inc., Austin, TX, USA). The primers were designed based on the Ensembl database’s chicken coding region, as displayed in Table 2. The *RPL32* and *HSP70* genes were used to normalize the results. The qRT-PCR was performed in triplicate with the SYBR Premix Ex TaqTM reagent Kit (TAKARA, Kusatsu, Japan) using the QuantStuio 7 Flex RealTime PCR System (Applied Biosystems, 133 Massachusetts, America). The amplification protocol was as follows: 95 °C for 3 min, followed by 40 cycles of 95 °C for 3 s and annealing temperature for 34 s, as mentioned previously [16]. The data were calculated using the 2^−△△CT^ method [34].

### 2.5. Statistical Analysis

The present experiment was designed based on a completely randomized design. The general linear model by two-way ANOVA, 2 × 2 and, T-test for (shear force (g), drip loss (%) and gene expression data) were applied to analyze the data using SPSS 26 (IBM). The model of two-way ANOVA included the main effects of treatments (control or MET), the gender (male or female) and interactions between treatments and gender. The means were compared by using Tukey’s range test to determine significant differences among means, differences were considered significant at a value of *p* < 0.05. The lower cases (^a/b^) represent significant differences within the main effects of treatments while upper cases (^A/B^) represent significant differences within the main effects of gender.

## 3. Results

### 3.1. Effects of Paternal Methionine on Carcass Traits of Progeny

The results of progeny carcass traits are shown in Table 3. Although the heavier live body weight at day 49 of control group compared to MET group (*p* < 0.05), the eviscerated weight was not different between the two groups. The higher relative eviscerated yield and relative thigh muscle % were observed in progeny from the MET group (*p* < 0.05 and *p* < 0.05, respectively). The relative abdominal fat was lower in MET group (*p* < 0.01) and no differences in relative breast muscles % between the progeny. The carcass characteristics were significantly increased (*p* < 0.01) in males, while no significant interaction between treatments and gender was observed.

### 3.2. Effects of Paternal Methionine on Meat Quality of Progeny

The results of breast and thigh muscles pH-value and color are presented in Table 4 and Table 5, respectively. The lightness (L*45 min and 24 h) and redness (a*45 min and 24 h) in breast muscles of the MET group were higher (*p* < 0.05), while the redness (a*_24 h_) and yellowness (b*_24 h_) in thigh muscles of MET group were higher (*p* < 0.05). A higher pH_24 h_ in breast and thigh muscles was observed (*p* < 0.01) in the MET group, and no differences in other indicators. Males displayed higher meat redness (a*) *p* < 0.01). The male’s thigh and breast muscles drip loss (%) and shear force (g) as presented in Table 6 were improved (*p* < 0.01 and *p* < 0.05, respectively) in MET group. There were no diet × gender interaction for any of the meat quality measures.

### 3.3. Effects of Methionine on Gene Expression of Breast and Thigh Muscles of Male’s Progeny

**Figure 1:**
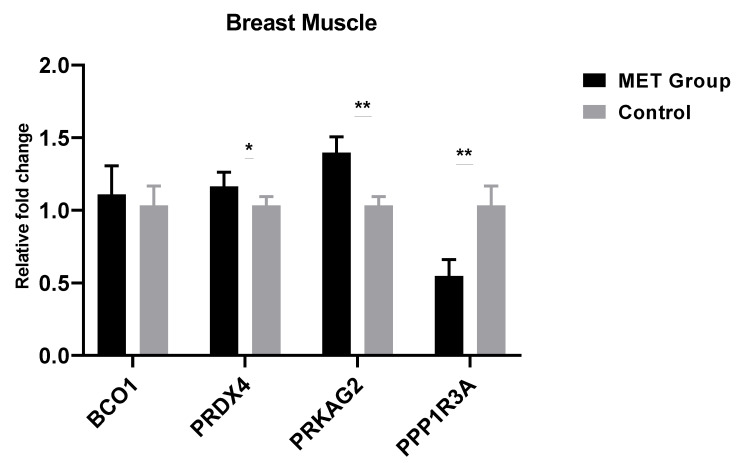
Breast muscle relative fold change (gene expression) of male progeny with paternal dietary Met. Control group (0.28% Met), Methionine group (0.38% Met as 0.28% Met + 0.1% coated Met). The data are expressed as mean ± SEM and n = 12. Significance (*: *p* < 0.05; **: *p* < 0.01). *BCO1*, beta-carotene oxygenase 1; *PRDX4*, peroxiredoxin 4; *PRKAG2*, protein kinase AMP-activated non-catalytic subunit gamma 2; *PPP1R3A*, protein phosphatase 1 regulatory subunit 3A.

**Figure 2:**
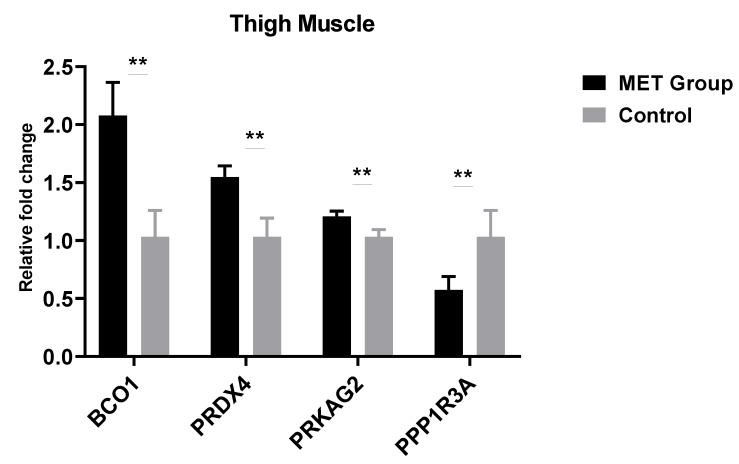
Thigh muscle relative fold change (gene expression) of male progeny with paternal dietary Met. Control group (0.28% Met), Methionine group (0.38% Met as 0.28% Met + 0.1% coated Met). The data are expressed as mean ± SEM and n = 12. Significance (**: *p* < 0.01). *BCO1*, beta-carotene oxygenase 1; *PRDX4*, peroxiredoxin 4; *PRKAG2*, protein kinase AMP-activated non-catalytic subunit gamma 2; *PPP1R3A*, protein phosphatase 1 regulatory subunit 3A.

To verify the effect of methionine level on progeny’s meat quality traits, we analyzed the expression of four genes associated with meat quality. The relative gene expression of breast and thigh muscles of male progeny are presented in Figure 1 and Figure 2 Figure 1; Figure 2, respectively. The expression level of *PRKAG2* and *PRDX4* genes positively related to pH-value were up-regulated significantly (*p* < 0.01) in breast and thigh muscles of MET group. In contrast, the expression of *PPP1R3A* pH-value negatively related gene down-regulated significantly (*p* < 0.01), consistent with pH-value. The expression levels of *BCO1* related to meat color, was up-regulated in the MET group thigh muscle and, consistent with the b*-value.

## 4. Discussion

Results from the current study demonstrate that paternal 0.1% Met dietary supplementation, positively affects the relative eviscerated carcass yield and relative thigh muscle yield in the progeny, while the live body weight was negatively affected. These findings are in accordance with previous research [35,36,37], which confirmed that methionine level enhanced broilers carcass and thigh weight. Methionine has a vital role in protein metabolism and muscle growth [38] having a role in muscle creatinine synthesis. Muscle development may be more sensitive to coated Met inclusion because of its’ higher absorption rate [39]. The reduction in relative abdominal fat observed in the Met group, is supported by previous studies where there is a significant drop in abdominal fat of broilers supplemented with dietary Met [40,41,42]. This decrease is possibly attributed to increased carnitine synthesis in liver and the abdominal fat hormonal sensitive lipase activity [42].

The results regarding meat quality indicated that the coated Met supplementation has a significant positive effect. The pH value has a vital effect on meat quality, influencing the attributes responsible for processing suitability, nutritional properties, and shelf life of meat [43]. Met supplementation resulted in higher pH_24h_ values, as reported in former studies [44,45,46]. Tenderness has a significant impact on the meat’s consumer preference, and the dietary Met decreased shear force. Similar findings were reported in former studies [16,45]. Drip loss is considered a relevant indicator for determining the quality of meat where excessive drip loss is not desired. Drip loss (%) decreased in MET group, consistent with previous studies reported that increasing Met supplementation positively impact drip loss in broiler meat [44,46,47]. Meat color is a characteristic of importance to consumer acceptability. In general, dietary Met supplementation led to higher lightness and redness values in breast muscles and redness in thigh muscles, with similar results also observed in previous studies [16,46]. There is a fluctuation in the meat color values between the thigh and breast muscles of different groups, all were in the normal range for measures of good meat quality. The meat color is highly affected by conditions of slaughter, diet, and genetic [3,48].

To verify the meat quality results, we determined the gene expression rates of four genes associated with meat quality. The results revealed that the expression of *PRDX4* and *PRKAG2* in the muscles of Met group were significantly up-regulated, while the *PPP1R3A* was significantly down-regulated, all changes consistent with the meat pH-values. The PRDX4 gene has an essential function in regulating the pH-value of chicken breast muscles by catalyzing hydrogen peroxide reduction and positively correlated with the pH-value [49,50]. *PRKAG2* affects glycogen metabolism in muscle by activating the AMP signaling pathway, and is inversely related to muscle glycogen metabolism that, is responsible for lower pH in muscles [51]. The glycogen storage decrease in the chickens breast muscles tends to be associated with lower expression of *PPP1R3A* [52].

In our experiment, the *BCO1* expression is up-regulated in thigh muscles of MET group and is consistent with increased yellowness (b*), because *BCO1* expression is consistent with the cumulation of carotenoids affecting the meat yellow color by changing the yellow pigments lutein and zeaxanthin content [53,54].

A better progeny performance is achieved when they come from roosters with higher semen quality [55]. Epigenetics studies on male reproduction indicate that the effect of nutritional and environmental influences on the health of progeny is not limited to females. Still, it is also shared by males, where epigenetic sperm signatures (cellular memory) are passed on to oocytes and therefore, can influence the development of embryos and progeny [56]. Epigenetic differences may occur during the “dietary transition” phase when the animals subjected to an unbalanced nutritional diet may influence DNA methylation. [57]. By contributing methyl groups to cytosine methylation, Met is essential to epigenetic reactions [58]. The status of CpG methylation, hepatic lipid metabolism-related gene expression, and growth performance could be altered by changing dietary methionine levels [59]. The epigenetic marks are inheritable and capable of turning genes on or off in progeny. Despite this, there is still little understanding of transgenerational epigenetics in animals [25].

## 5. Conclusions

In conclusion, paternal dietary supplementation with 0.1% methionine could improve progeny carcass traits including increased eviscerated carcass and thigh muscle yields and reducing abdominal fat content, also meat quality characteristics such as drip loss, shear force, pH value, and color. Further research is required to explore the mechanism(s) of transgenerational effects of paternal nutritional supplementation on progeny.

## Figures and Tables

**Table 1:** Ingredient and calculated nutrient composition of experimental diet for parental birds ^1^.

Item	Control Group	MET Group
Ingredient (%)		
Corn	65.37	65.37
Wheat bran	15.00	15.00
Soybean meal, 44% CP	13.60	13.50
Soybean oil	2.00	2.00
Limestones	1.50	1.50
Dicalcium phosphate	1.23	1.23
Salt	0.30	0.30
Coated methionine	0.00	0.10
Premix ^2^	1.00	1.00
Total	100.00	100.00
Calculated composition		
ME (Mcal/kg)	2.87	2.87
CP (%)	13.82	13.82
Ca (%)	0.94	0.94
TP (%)	0.61	0.61
Met (%)	0.28	0.38
Analyzed composition		
CP (%)	13.85	13.89
Met (%)	0.29	0.40

^1^ Control group (0.28% Met), Methionine group (0.38% Met as 0.28% Met + 0.1% coated Met). ^2^ Provides per kg of diet: 10,400 IU vit. A; 2500 IU vit. D3; 30 IU vit. E; 2 mg vit. K3; 2 mg vit. B1; 8.5 mg vit. B2; 4 mg vit. B6; 0.015 mg vit. B12; 2 mg biotin; 3 mg folic acid; 35 mg niacin; 400 mg choline chloride; 40 mg calcium pantothenate; 8 mg Cu; 65 mg Zn; 80 mg Fe; 1 mg I; 80 mg Mn; and 0.3 mg Se.

**Table 2:** A list of the genes and primer sequences.

Accession Numbers	Genes ^1^	Primers	Amplicon Size (bp)
XM_015281206.2	*PRKAG2*	F:5’-TGCCTTCATACATCCAGACACTCCTAT-3’	279
		R:5’-ACCTCAGCCTTCACTATCCTATCAACA-3’	
XM_416800.6	*PRDX4*	F:5’-CCACCCTAGCCATGGATTACC-3’	197
		R:5’-AGGCATGGCTACATCTTCGAG-3’	
XM_015292519.2	*BCO1*	F:5’-TCCAACTTCCGCAACTGCTGTA-3’	314
		R:5’-TTGGCTCAGACACCACAACACA-3’	
XM_004937541.2	*PPP1R3A*	F:5’-TGAACGGCATTATACGAGTCCTCAA-3’	195
		R:5’-ATTCCACTTTGGCTCCATCTCTCTG-3’	
XM_015293128.2	*RPL32*	F:5’-AGTTCATCCGCCACCAGTCTGAT-3’	147
		R:5’-GCTTCGTCTTCTTGTTGCTCCCATA-3’	
NM_001006685.1	*HSP70*	F:5’-TCTGCTCCTGTTGGATGTC-3’	95
		R:5’-TGGGAATGGTGGTGTTACG-3’	

^1^ F: forward; R: reverse; *PRDX4*: peroxiredoxin 4; *PPP1R3A*: protein phosphatase 1 regulatory subunit 3A; *PRKAG2*: protein kinase AMP-activated non-catalytic subunit gamma 2; *BCO1*: beta-carotene oxygenase 1; *HSP70*: heat shock 70 kDa protein 2; *RPL32*: ribosomal protein L32.

**Table 3:** Effects of paternal dietary Met on carcass characteristics of progeny ^1, 2, 3^.

Items	Treatments		Gender		SEM		Significance		
	Control	MET Group	Male	Female		Treatments		Gender	Interaction
Live weight(g)	2002.5 ^a^	1942.9 ^b^	2009.8 ^A^	1871.7 ^B^	17.3	*		**	NS
Eviscerated weight(g)	1631.0	1600.0	1663.1 ^A^	1516.4 ^B^	14.9	NS		**	NS
Eviscerated yield (%)	81.47 ^b^	82.33 ^a^	82.79 ^A^	81.01 ^B^	0.22	*		**	NS
Breast muscle (%)	26.65	26.41	26.10 ^A^	26.97 ^B^	0.14	NS		**	NS
Thigh muscle (%)	29.09 ^b^	29.99 ^a^	29.74 ^A^	29.03 ^B^	0.09	**		**	NS
AF (%)	0.97 ^a^	0.88 ^b^	0.83 ^B^	1.02 ^A^	0.02	**		**	NS

^1^ Control group (0.28% Met), Methionine group (0.38% Met as 0.28% Met+0.1% coated Met). ^2^ The data are expressed as the mean and pooled SEM of 3 replicates per group and n = 20 males and 23 females. The lower cases (^a/b^) represent significant differences within the main effects of treatments while upper cases (^A/B^) represent significant differences within the main effects of gender. ^3^ where * represents *p* < 0.05, while ** is *p* < 0.01 and NS is non-significant.

**Table 4:** Effects of paternal dietary Met on breast muscles quality of progeny ^1, 2, 3^.

Items ^4^	Treatments		Gender		SEM	Significance		
	Control	MET Group	Male	Female		Treatments	Gender	Interaction
L*45 min	47.53 ^b^	48.13 ^a^	47.85	47.81	0.10	**	NS	NS
a*45 min	1.82 ^b^	2.13 ^a^	2.09 ^A^	1.87 ^B^	0.04	**	**	NS
b*45 min	3.75	3.99	3.84	3.82	0.05	NS	NS	NS
pH45 min	6.45	6.43	6.43	6.45	0.01	NS	NS	NS
L*24 h	54.43 ^b^	55.39 ^a^	55.03	54.81	0.14	*	NS	NS
a*24 h	2.19 ^b^	2.31 ^a^	2.48 ^A^	2.02 ^B^	0.06	*	**	NS
b*24 h	6.19	6.10	6.33	5.97	0.09	NS	NS	NS
pH24 h	5.81 ^b^	5.99 ^a^	5.87	5.84	0.01	**	NS	NS

^1^ Control group (0.28% Met), Methionine group (0.38% Met as 0.28% Met+0.1% coated Met). ^2^ The data are expressed as the mean and pooled SEM of 3 replicates per group and n = 15 males or females. The lower cases (^a/b^) represent significant differences within the main effects of treatments while upper cases (^A/B^) represent significant differences within the main effects of gender. ^3^ where * represents *p* < 0.05, while ** is *p* < 0.01 and NS is non-significant. ^4^ a*: redness; L*: lightness; b*: yellowness; 45 min: 45 min after slaughter; 24 h: 24 h after slaughter.

**Table 5:** Effects of paternal dietary Met on thigh muscles quality of progeny ^1, 2, 3^.

Item ^4^	Treatments		Gender		SEM	Significance		
	Control	MET Group	Male	Female		Treatments	Gender	Interaction
L*45 min	52.14	52.19	52.28	52.05	0.18	NS	NS	NS
a*45 min	3.44	3.39	3.57^A^	3.26^B^	0.06	NS	**	NS
b*45 min	1.63	1.69	1.56	1.76	0.07	NS	NS	NS
pH45 min	6.45	6.44	6.45	6.44	0.01	NS	NS	NS
L*24 h	54.36	54.73	54.53	54.55	0.17	NS	NS	NS
a*24 h	3.22 ^b^	3.51 ^a^	3.45	3.28	0.05	**	NS	NS
b*24 h	2.75 ^b^	3.32 ^a^	3.01	3.06	0.07	**	NS	NS
pH24 h	6.22 ^b^	6.40 ^a^	6.29	6.27	0.01	**	NS	NS

^1^ Control group (0.28% Met), Methionine group (0.38% Met as 0.28% Met+0.1% coated Met). ^2^ The data are expressed as the mean and pooled SEM of 3 replicates per group and n = 15 males or females. The lower cases (^a/b^) represent significant differences within the main effects of treatments while upper cases (^A/B^) represent significant differences within the main effects of gender. ^3^ where * represents *p* < 0.05, while ** is *p* < 0.01 and NS is non-significant. ^4^ a*: redness; L*: lightness; b*: yellowness; 45 min: 45 min after slaughter; 24 h: 24 h after slaughter.

**Table 6:** Effects of paternal dietary Met on drip loss and shear force of male progeny ^1, 2, 3^.

Items	Control	MET Group	SEM	Significance
Breast drip loss (%)	3.68 ^a^	3.47 ^b^	0.12	*
Thigh drip loss (%)	3.64 ^a^	2.71 ^b^	0.11	**
Breast shear force(g)	3028.1 ^a^	2543.0 ^b^	56.0	**
Thigh shear force(g)	1616.5 ^a^	1465.0 ^b^	76.8	*

^1^ Control group (0.28% Met), Methionine group (0.38% Met as 0.28% Met+0.1% coated Met). ^2^ The data are expressed as the mean ± SEM and n = 12. ^3^ where * represents *p* < 0.05, while ** is *p* < 0.01 and The lower cases (a/b) represent significant differences.

## References

[1] Fanatico A.C., Pillai P.B., Emmert J.L., Owens C.M. (2007). Meat quality of slow- and fast-growing chicken genotypes fed low-nutrient or standard diets and raised indoors or with outdoor access. Poult. Sci..

[2] Dransfield E., Sosnicki A.A. (1999). Relationship between muscle growth and poultry meat quality. Poult. Sci..

[3] Fletcher D.L. (2002). Poultry meat quality. World Poult. Sci. J..

[4] Zhao J.P., Zhao G.P., Jiang R.R., Zheng M.Q., Chen J.L., Liu R.R., Wen J. (2012). Effects of diet-induced differences in growth rate on metabolic, histological, and meat-quality properties of 2 muscles in male chickens of 2 distinct broiler breeds. Poult. Sci..

[5] Drażbo A., Kozłowski K., Chwastowska-Siwiecka I., Sobczak A., Kwiatkowski P., Lemme A. (2015). Effect of different dietary levels of DL-methionine and the calcium salt of DL- 2-hydroxy-4-[methyl] butanoic acid on the growth performance, carcass yield and meat quality of broiler chickens. Eur. Poult. Sci..

[6] Wen C., Jiang X.Y., Ding L.R., Wang T., Zhou Y.M. (2017). Effects of dietary methionine on growth performance, meat quality and oxidative status of breast muscle in fast- and slow-growing broilers. Poult. Sci..

[7] Alagawany M., Abd El-Hack M.E., Arif M., Ashour E.A. (2016). Individual and combined effects of crude protein, methionine, and probiotic levels on laying hen productive performance and nitrogen pollution in the manure. Environ. Sci. Pollut. Res. Int..

[8] Bunchasak C. (2009). Role of dietary methionine in poultry production. J. Poult. Sci..

[9] Niculescu M.D., Zeisel S.H. (2002). Diet, methyl donors and DNA methylation: Interactions between dietary folate, methionine and choline. J. Nutr..

[10] Schutte J.B., De Jong J., Smink W., Pack M. (1997). Replacement value of betaine for DL-methionine in male broiler chicks. Poult. Sci..

[11] Binder M. (2003). Life cycle analysis of DL-methionine in broiler meat production. Amino News.

[12] Elwan H.A.M., Elnesr S.S., Xu Q., Xie C., Dong X., Zou X. (2019). Effects of In Ovo Methionine-Cysteine Injection on Embryonic Development, Antioxidant Status, IGF-I and TLR4 Gene Expression, and Jejunum Histomorphometry in Newly Hatched Broiler Chicks Exposed to Heat Stress during Incubation. Animals (Basel).

[13] Nockels C. (1979). Protective effects of supplemental vitamin E against infection. Fed. Proc..

[14] Gao J., Lv Z., Li C., Yue Y., Zhao X., Wang F., Guo Y. (2014). Maternal zinc supplementation enhanced skeletal muscle development through increasing protein synthesis and inhibiting protein degradation of their offspring. Biol. Trace. Elem. Res..

[15] Guerrero-Bosagna C., Skinner M.K. (2012). Environmentally induced epigenetic transgenerational inheritance of phenotype and disease. Mol. Cell Endocrinol..

[16] Liu R., Tan X., Zhao G., Chen Y., Zhao D., Li W., Zheng M., Wen J. (2020). Maternal dietary methionine supplementation influences egg production, and the growth performance and meat quality of offspring. Poult. Sci..

[17] Ruan D., Fouad A.M., Fan Q., Xia W., Wang S., Chen W., Lin C., Wang Y., Yang L., Zheng C. (2018). Effects of dietary methionine on productivity, reproductive performance, antioxidant capacity, ovalbumin and antioxidant-related gene expression in laying duck breeders. Br. J.Nutr..

[18] Kalvandi O., Sadeghi A., Karimi A. (2019). Methionine supplementation improves reproductive performance, antioxidant status, immunity and maternal antibody transmission in breeder Japanese quail under heat stress conditions. Arch. Anim. Breed..

[19] Hayat Z., Rehman A.U., Akram K., Farooq U., Saleem G. (2015). Evaluation of a natural methionine source on broiler growth performance. J. Sci. Food. Agric..

[20] Wen C., Jiang X., Ding L., Wang T., Zhou Y. (2017). Effects of dietary methionine on breast muscle growth, myogenic gene expression and IGF-I signaling in fast- and slow-growing broilers. Sci. Rep..

[21] Zhang S., Saremi B., Gilbert E.R., Wong E.A. (2017). Physiological and biochemical aspects of methionine isomers and a methionine analogue in broilers. Poult. Sci..

[22] Conde-Aguilera J.A., Cholet J.C., Lessire M., Mercier Y., Tesseraud S., van Milgen J. (2016). The level and source of free-methionine affect body composition and breast muscle traits in growing broilers. Poult. Sci..

[23] Alam M.S., Teshima S.-I., Koshio S., Ishikawa M., Uyan O., Hernandez L.H.H., Michael F.R. (2005). Supplemental effects of coated methionine and/or lysine to soy protein isolate diet for juvenile kuruma shrimp, Marsupenaeus japonicus. Aquaculture.

[24] Smith S., Boling J. (1984). Lipid coating as a mode of protecting free methionine from ruminal degradation. J. Anim. Sci..

[25] Triques G.E., Cristo A.B.D., Canevese M., Marques P.F.D.S., Burin Junior A.M., Fernandes J.I.M. (2019). Effect of Antioxidant Supplementation in Diets of Roosters during the Post-Peak Phase on Reproduction and Production Characteristics of Offspring. Ciênc. Anim. Bras..

[26] Alderey A.A., Nasr A.M.E., Abu khashaba H.A., Samak H.R. (2019). Performance of Gimmizah Cockerels Fed Two Levels of Methionine and Lysine and Two Levels of Protein. J. Anim. Poult. Prod. Mansoura Univ..

[27] Shanmugam M., Prakash B., Rao S.V.R., Panda A.K. (2016). Effect of dietary energy and crude protein on semen parameters and fertility in layer breeders males. Indian J. Anim. Sci..

[28] Gómez-Polo C., Montero J., Gómez-Polo M., Martin Casado A. (2020). Comparison of the CIELab and CIEDE 2000 color difference formulas on gingival color space. J. Prosthodont.

[29] O’brien W., Groh C., Boenke K. (1990). A new, small-color-difference equation for dental shades. J. Dent. Res..

[30] Sun Y., Zhao G., Liu R., Zheng M., Hu Y., Wu D., Zhang L., Li P., Wen J. (2013). The identification of 14 new genes for meat quality traits in chicken using a genome-wide association study. BMC Genomics.

[31] Wang X., Chen X., Tan H., Zhang D., Zhang H., Wei S., Yan H.C. (2013). Nutrient density and slaughter age have differential effects on carcase performance, muscle and meat quality in fast and slow growing broiler genotypes. Poult. Sci..

[32] Devatkal S.K., Vishnuraj M.R., Kulkarni V.V., Kotaiah T. (2018). Carcass and meat quality characterization of indigenous and improved variety of chicken genotypes. Poult. Sci..

[33] Zhang Y., Liu Z., Liu R., Wang J., Zheng M., Li Q., Cui H., Zhao G., Wen J. (2018). Alteration of Hepatic Gene Expression along with the Inherited Phenotype of Acquired Fatty Liver in Chicken. Genes (Basel).

[34] Livak K.J., Schmittgen T.D.J.m. (2001). Analysis of relative gene expression data using real-time quantitative PCR and the 2^−ΔΔCT^ method. Methods.

[35] Yoo J., Yi Y.J., Wickramasuriya S.S., Kim E., Shin T.K., Cho H.M., Kim N., Heo J.M. (2017). Evaluation of sulphur amino acid requirement of male Korean native ducklings from hatch to 21 day of age. Br. Poult. Sci..

[36] Majdeddin M., Golian A., Kermanshahi H., Michiels J., De Smet S. (2019). Effects of methionine and guanidinoacetic acid supplementation on performance and energy metabolites in breast muscle of male broiler chickens fed corn-soybean diets. Br. Poult. Sci..

[37] Rehman A.U., Arif M., Husnain M.M., Alagawany M., Abd El-Hack M.E., Taha A.E., Elnesr S.S., Abdel-Latif M.A., Othman S.I., Allam A.A. (2019). Growth Performance of Broilers as Influenced by Different Levels and Sources of Methionine Plus Cysteine. Animals (Basel).

[38] Hickling D., Guenter W., Jackson M.E. (1990). The effects of dietary methionine and lysine on broiler chicken performance and breast meat yield. Can. J. Anim. Sci..

[39] Lu J., Hua Y., Fu W.-Z., Zhou F., Yang B.-B., Xiao J.-X., Liu M.-H., Shao Q.-J. (2014). Effects of supplementation coated lysine and methionine in mixture protein diets on growth performance, digestibility and serum biochemical indices of juvenile black sea bream, Acanthopagrus schlegelii. Turk. J. Fish. Aquat. Sci..

[40] Mandal A., Elangovan A., Johri T.S. (2004). Comparing bio-efficacy of liquid DL-methionine hydroxy analogue free acid with DL-methionine in broiler chickens. Asian Austral. J. Anim..

[41] Liu Y., Song G., Yi G., Hou Y., Huang J., Knight C.D. (2006). Effect of supplementing 2-hydroxy-4-(methylthio) butanoic acid and DL-methionine in corn-soybean-cottonseed meal diets on growth performance and carcass quality of broilers. Asian Austral. J. Anim..

[42] Zhan X.A., Li J.X., Xu Z.R., Zhao R.Q. (2006). Effects of methionine and betaine supplementation on growth performance, carcase composition and metabolism of lipids in male broilers. Br. Poult. Sci..

[43] Woelfel R.L., Owens C.M., Hirschler E.M., Martinez-Dawson R., Sams A.R. (2002). The characterization and incidence of pale, soft, and exudative broiler meat in a commercial processing plant. Poult. Sci..

[44] Albrecht A., Herbert U., Miskel D., Heinemann C., Braun C., Dohlen S., Zeitz J.O., Eder K., Saremi B., Kreyenschmidt J. (2017). Effect of methionine supplementation in chicken feed on the quality and shelf life of fresh poultry meat. Poult. Sci..

[45] Zonenberg Ł., Drażbo A. (2018). The effect of increased methionine in broiler chicken diets on the quality of breast muscles at different times of vacuum storage under refrigeration. Sci. Ann. Pol. Soc. Anim. Prod..

[46] Albrecht A., Hebel M., Heinemann C., Herbert U., Miskel D., Saremi B., Kreyenschmidt J. (2019). Assessment of Meat Quality and Shelf Life from Broilers Fed with Different Sources and Concentrations of Methionine. J. Food Qual..

[47] Wang Z.G., Pan X.J., Peng Z.Q., Zhao R.Q., Zhou G.H. (2009). Methionine and selenium yeast supplementation of the maternal diets affects color, water-holding capacity, and oxidative stability of their male offspring meat at the early stage. Poult. Sci..

[48] Mugler D., Cunningham F.E. (1972). Factors affecting poultry meat color—A review. World Poult. Sci. J..

[49] Li X., Liu X., Nadaf J., Le Bihan-Duval E., Berri C., Dunn I., Talbot R., De Koning D.-J. (2015). Using targeted resequencing for identification of candidate genes and SNPs for a QTL Affecting the pH value of chicken meat. G3 Genes Genomes Genet..

[50] Nadaf J., Berri C., Dunn I., Godet E., Le Bihan-Duval E., De Koning D.J. (2014). An expression QTL of closely linked candidate genes affects pH of meat in chickens. Genetics.

[51] Sibut V., Hennequet-Antier C., Le Bihan-Duval E., Marthey S., Duclos M.J., Berri C. (2011). Identification of differentially expressed genes in chickens differing in muscle glycogen content and meat quality. BMC Genomics.

[52] Abasht B., Zhou N., Lee W.R., Zhuo Z., Peripolli E. (2019). The metabolic characteristics of susceptibility to wooden breast disease in chickens with high feed efficiency. Poult. Sci..

[53] Jlali M., Graulet B., Chauveau-Duriot B., Chabault M., Godet E., Leroux S., Praud C., Le Bihan-Duval E., Duclos M.J., Berri C. (2012). A mutation in the promoter of the chicken β, β-carotene 15, 15′-monooxygenase 1 gene alters xanthophyll metabolism through a selective effect on its mRNA abundance in the breast muscle. J. Anim. Sci..

[54] Le Bihan-Duval E., Nadaf J., Berri C., Pitel F., Graulet B., Godet E., Leroux S.Y., Demeure O., Lagarrigue S., Duby C.J.P.O. (2011). Detection of a Cis eQTL controlling BMCO1 gene expression leads to the identification of a QTG for chicken breast meat color. PLoS ONE.

[55] Chang A., Halley J., Silva M. (2016). Can feeding the broiler breeder improve chick quality and offspring performance?. Anim. Prod. Sci..

[56] Schagdarsurengin U., Steger K. (2016). Epigenetics in male reproduction: Effect of paternal diet on sperm quality and offspring health. Nat. Rev. Urol..

[57] Zhang N. (2015). Epigenetic modulation of DNA methylation by nutrition and its mechanisms in animals. Anim. Nutr..

[58] Tehlivets O., Malanovic N., Visram M., Pavkov-Keller T., Keller W. (2013). S-adenosyl-L-homocysteine hydrolase and methylation disorders: Yeast as a model system. Biochim. Biophys. Acta.

[59] Zhang N. (2018). Role of methionine on epigenetic modification of DNA methylation and gene expression in animals. Anim. Nutr..

